# 

**Published:** 1896-07

**Authors:** 


TABLE OF CONTENTS ON LAST ADVERTISING PAGE.

VOL. XII. JULY, 1896. No. 1.

### TEXAS Medical Journal.

SUBSCRIPTION, $2.00 A YEAR, IN ADVANCE.
SINGLE COPIES, 25 CENTS.

PUBLISHED AT AUSTIN, TEXAS, BY
F. E. DANIEL, M. D., & S. E. HUDSON, M. D.,
EDITORS AND PROPRIETORS.

Eastern Agents: The Monlhan-Fairchild Special Agency, 21 Park Place, New York City.

Entered at the Postoffice at Austin, Texas, as Second-class Mail Matter.

